# Identification of 3-((4-Hydroxyphenyl)amino)propanoic Acid Derivatives as Anticancer Candidates with Promising Antioxidant Properties

**DOI:** 10.3390/molecules29133125

**Published:** 2024-06-30

**Authors:** Povilas Kavaliauskas, Birutė Grybaitė, Birute Sapijanskaite-Banevič, Kazimieras Anusevičius, Ilona Jonuškienė, Rima Stankevičienė, Rūta Petraitienė, Vidmantas Petraitis, Ramunė Grigalevičiūtė, Edita Meškinytė, Rolandas Stankevičius, Vytautas Mickevičius

**Affiliations:** 1 Department of Organic Chemistry, Kaunas University of Technology, LT-50254 Kaunas, Lithuania; birute.grybaite@ktu.lt (B.G.); birute.sapijanskaite@ktu.lt (B.S.-B.); kazimieras.anusevicius@ktu.lt (K.A.); ilona.jonuskiene@ktu.lt (I.J.); rima.stankeviciene@ktu.lt (R.S.); vytautas.mickevicius@ktu.lt (V.M.); 2 Division of Infectious Diseases, Department of Medicine, Weill Cornell Medicine of Cornell University, New York, NY 10065, USA; rop2016@med.cornell.edu (R.P.); vip2007@med.cornell.edu (V.P.); 3 Institute of Infectious Diseases and Pathogenic Microbiology, LT-59116 Prienai, Lithuania; 4 Biological Research Center, Lithuanian University of Health Sciences, LT-44307 Kaunas, Lithuania; ramune.grigaleviciute@lsmuni.lt; 5 Center for Discovery and Innovation, Hackensack Meridian Health, Nutley, NJ 07110, USA; 6 Department of Animal Nutrition, Lithuanian University of Health Sciences, LT-44307 Kaunas, Lithuania; rolandas.stankevicius@lsmuni.lt; 7 Center of Animal Production Research and Innovation, Agriculture Academy, Vytautas Magnus University, LT-44248 Kaunas, Lithuania; edita.meskinyte@vdu.edu

**Keywords:** 3-((4-hydroxyphenyl)amino)propanoic acid derivatives, anticancer, A549

## Abstract

Various cancer-associated morbidities remain a growing global health challenge, resulting in a significant burden on healthcare systems worldwide due to high mortality rates and a frequent lack of novel therapeutic options for advanced and localized disease. Reactive oxygen species (ROS) play an important role in cancer pathogenesis and response to chemotherapeutics; therefore, it is crucial to develop novel compounds with both antioxidant and anticancer activity. In this study, a series of previously reported 3-((4-hydroxyphenyl)amino)propanoic acid derivatives (compounds **1**–**36**) were evaluated for their anticancer and antioxidant activities. Compounds **12**, **20**–**22**, and **29** were able to reduce A549 cell viability by 50% and suppress A549 cell migration in vitro. These compounds also showed favorable cytotoxicity properties towards noncancerous Vero cells. The most promising candidate, compound **20**, exhibited potent antioxidant properties in the DPPH radical scavenging assay. These results demonstrate that 3-((4-hydroxyphenyl)amino)propanoic acid could be further explored as an attractive scaffold for the development of novel anticancer and antioxidant candidates.

## 1. Introduction

Various cancer-associated morbidities remain a growing global health challenge, resulting in a significant burden on healthcare systems worldwide due to its high mortality rates and often lack of novel therapeutic options targeting advanced and localized disease [1,2,3]. Despite advancements in cancer treatment strategies, the limited efficacy of current therapies, coupled with the emergence of treatment-resistant tumor sub-populations, underscores the critical need for novel anticancer compounds, targeting novel or multiple cancerous cell targets [4,5,6]. Particularly, challenging cancers such as lung cancer pose a significant clinical hurdle, with limited therapeutic options available, especially in advanced or metastatic disease stages [4,7,8]. Therefore, it is crucial to develop novel strategies targeting novel anticancer compounds for further preclinical evaluation [9,10,11,12]. 

Heterocyclic compounds, characterized by their diverse chemical structures and pharmacological activities, stand out as potential frameworks for the synthesis of such compounds [13,14,15]. Their inherent structural complexity and versatility make them attractive candidates for drug discovery efforts aimed at addressing the pressing need for effective anticancer agents [13,15]. Recognizing the vast chemical space offered by heterocyclic scaffolds, there arises a critical need to systematically screen existing compound libraries for compounds with untapped anticancer properties [13,16].

Among the versatile aromatic and heterocyclic scaffolds, phenol (4-hydroxyphenyl) emerges as a particularly promising pharmacophore for the development of anticancer compounds with antioxidant properties. Previous studies have demonstrated that compounds containing 4-hydroxyphenyl substituent show promising anticancer activity, targeting tubulin [17]. Another study reported that the generation of indole-based compounds with 4-hydroxyphenyl substituents results in broad-spectrum anticancer candidates [18]. This suggests that 4-hydroxyphenyl scaffold, especially incorporated into synthetically versatile scaffolds possesses distinctive physicochemical attributes that render it highly attractive for anticancer drug design. Furthermore, the presence of hydroxy groups within the molecular structure confers antioxidant activity, offering the opportunity to modulate oxidative stress, a pivotal driver of cancer progression and therapeutic resistance [19,20,21,22]. 

In our previous work, we reported the synthesis of 3-((4-hydroxyphenyl)amino)propanoic acid derivatives bearing diverse aromatic and heterocyclic substituents [23]. These compounds exhibited notable antibacterial and antifungal properties, demonstrating efficacy against multidrug-resistant bacterial and fungal pathogens [23]. Given the observed antifungal activity of the 3-((4-hydroxyphenyl)amino)propanoic acid derivatives, we postulated their potential as anticancer agents, exploiting shared biochemical pathways across eukaryotic organisms. Moreover, the inclusion of 4-hydroxyphenyl moieties within these compounds suggested inherent antioxidant properties, rendering this novel class of compounds compelling candidates for early-stage anticancer drug discovery. In this study, we report the screening anticancer and antioxidant properties of 3-((4-hydroxyphenyl)amino)propanoic acid derivatives containing aromatic and heterocyclic substituents. 

## 2. Results

### 2.1. 3-((4-Hydroxyphenyl)amino)propanoic Acid Derivatives Demonstrated Structure-Dependent Anticancer Activity

In our previous research, we described synthetic procedures to generate a series of 3-((4-hydroxyphenyl)amino)propanoic acid derivatives (compounds **1**–**36**) (Table 1) and characterized their antimicrobial properties using WHO priority pathogens [23]. 

**Figure 1:**
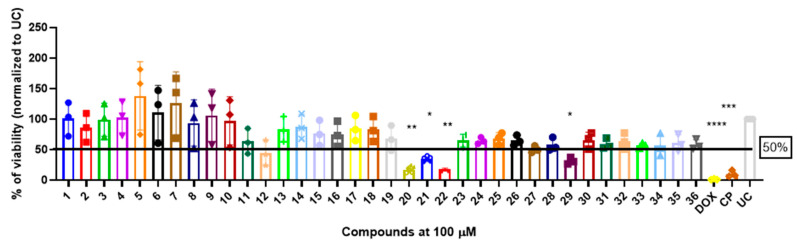
The (4-hydroxyphenyl)amino)propanoic acid derivatives **1**–**36** are able to induce the cytotoxicity in A549 cancerous cells. The cells were exposed with compounds **1**–**36** or doxorubicin (DOX) and cisplatin (CP) for 24 h and the viability was determined using MTT assay and normalized to untreated control (UC). The data are expressed as mean ± standard deviation of tree experimental replicas. The significance of the data was determined using a one-way ANOVA test. * *p* < 0.05, ** *p* < 0.01, *** *p* < 0.0002, **** *p* < 0.0001.

To evaluate the in vitro cytotoxic properties of compounds **1**–**36** (Table 1), we used a well-characterized A549 non-small cell lung cancer (NSCLC) cell culture model and compared the compound-mediated cytotoxicity with that of doxorubicin (DOX) and cisplatin (CP), standard chemotherapeutic agents used in NSCLC treatment (Figure 1).

The starting compound **1** demonstrated no noticeable in vitro anticancer activity. *N*-(4-hydroxyphenyl)-*β*-alanine hydrazide (compound **2**) reduced A549 cell viability to 86.1%. Compounds **3** (di propanoic acid), **4** (dimethyl ester), and **5** (dihydrazide) showed no noticeable anticancer activity. Hydrazones-type compounds **6**–**12** demonstrated variable cytotoxic activity. Among the tested hydrazones **6**–**12**, only compound **12**, bearing a 1-naphthyl substituent, exhibited anticancer activity, reducing A549 cell viability to 42.1% (Figure 1). Similar anticancer activity expressed by hydrazones have been previously reported in other studies [24,25,26,27]. The incorporation of aromatic substituents in hydrazone scaffolds greatly increases the anticancer activity in substituent-depended manner [28]. 

The introduction of various heterocyclic substituents in hydrazones (compounds **13**–**16**) resulted in enhanced anticancer activity, with these compounds reducing A549 cell viability to 75–83%. Interestingly, compounds containing an indolinone moiety, specifically compounds **17** and **18**, showed similar anticancer activity, with viability reductions of 84.6% and 82.9%, respectively. On the other hand, oxadiazole compound **19** demonstrated favorable anticancer activity, reducing A549 cell viability to 66.2% (Figure 1).

Compounds **20**–**22**, which contain substituted heterocyclic moieties, exhibited the most promising anticancer activity against A549 cells. Compound **20**, containing a 2-furyl substituent, significantly reduced A549 cell viability to 17.2% (*p* = 0.0011) in comparison to untreated control (UC). The incorporation of a 2-thienyl substituent (compound **21**) altered cytotoxic activity, reducing A549 viability to 34.2% (*p* = 0.0206) in comparison to UC. Replacing thiophene with 5-nitro-2-thienyl (compound **22**) restored anticancer activity, reducing A549 viability to 17.4% (*p =* 0.0012) in comparison to UC (Figure 1). Both 2-furyl and nitrothiophene substituents have been shown to be a strong pharmacophore harboring cytotoxic properties [29,30]. 

Hydrazones **25**–**27** demonstrated similar anticancer activity against A549 cells, with viability reductions ranging from 52.4% to 68.7%. Compound **28**, containing a phenyl substituent, reduced A549 cell viability to 57.8%. Compound **29**, with a 4-NO_2_ substitution on the phenyl group, showed favorable anticancer activity, reducing A549 viability to 31.2%. Other substitutions on the phenyl group, such as the incorporation of 4-Cl (compound **30**), diethylamino (compound **31)**, or 4-OH (compound **32**), resulted in similar anticancer activity, reducing A549 viability to 58.9–65.2%. Further incorporation of different heterocycles or aromatic substituents (compounds **33**–**36**) did not result in significant anticancer activity, as they were unable to reduce A549 viability by 50% (Figure 1).

**Figure 2:**
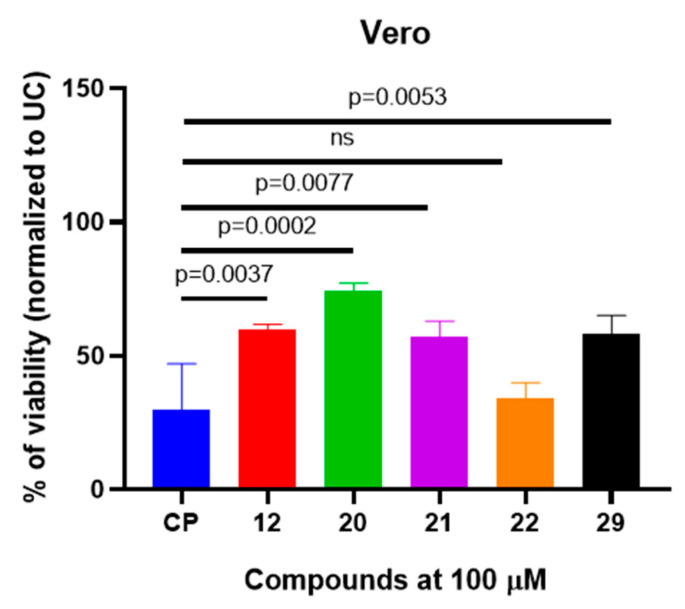
The selected (4-hydroxyphenyl)amino)propanoic acid derivatives demonstrated reduced cytotoxicity in noncancerous Vero cell culture model. The cells were exposed with compounds or cisplatin (CP) for 24 h and the viability was determined using MTT assay and normalized to untreated control (UC). The data are expressed as mean ± standard deviation of three experimental replicas. The significance of the data was determined using a one-way ANOVA test. ns—no significance.

After performing the initial screening and selecting the most potent compounds that reduce A549 cell viability by 50%, we selected compounds **12**, **20**–**22**, and **29** for further cytotoxicity characterization. To determine whether the observed cytotoxicity in A549 cells is specific to cancerous cells, we used noncancerous Vero cells for the cytotoxicity evaluation. Treatment with compounds **12** and **20**, **21**, and **29** resulted in significantly higher Vero cell viability compared to the cisplatin-treated Vero cells (*p* < 0.05) (Figure 2). Compound **22** showed no significant difference in Vero cell viability reduction compared to cisplatin, suggesting that compound-**22**-induced cytotoxicity to noncancerous Vero cells is similar to cytotoxicity induced by cisplatin. 

**Figure 3:**
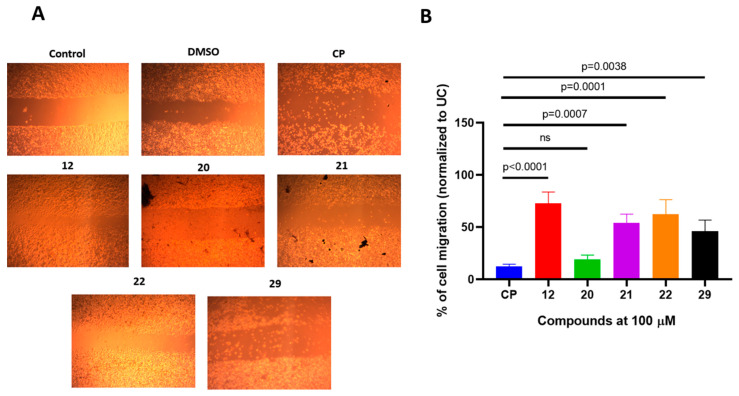
Selected (4-hydroxyphenyl)amino)propanoic acid derivatives are able to suppress the A549 migration in structure-dependent manner. Panel (**A**) shows representative photomicrograph images of the A549 monolayers undergoing mechanical damage (control), as well as A549 cell migration after 24-h treatment with DMSO or test compounds. Panel (**B**) demonstrates % of migrated A549 after measuring the area using Image J software (Version 1.51, 23 April 2018). The data are expressed as mean ± standard deviation of tree experimental replicas. The significance of the data was determined using a one-way ANOVA test. ns—no significance.

After selecting the most promising anticancer candidates among the 3-((4-hydroxyphenyl)amino)propanoic acid derivatives, we evaluated whether these compounds could suppress A549 cell migration. Cancerous cell migration is paramount for systemic multi-organ spread; therefore, novel compounds should contain properties that could reduce cancerous cell migration. To do so, we employed a well-established cell migration assay following mechanical disruption of the cell monolayer, followed by treatment with a fixed concentration of the test compounds (Figure 3A,B). All selected compounds significantly reduced A549 cell migration compared to the DMSO-treated control (*p* < 0.05). Cisplatin (CP) induced significantly higher suppression of A549 migration than compounds **12**, **21**, and **22**. Furthermore, compound **20** demonstrated the most promising A549 migration suppression activity, which was comparable to that of CP.

These findings highlight the potential of 3-((4-hydroxyphenyl)amino)propanoic acid derivatives as effective anticancer agents with selective cytotoxicity towards cancer cells and the ability to inhibit cancer cell migration.

### 2.2. 3-((4-Hydroxyphenyl)amino)propanoic Acid Derivatives ***1***–***36*** Shows Promising Antioxidant Properties

Oxidative stress, characterized by an imbalance between reactive oxygen species (ROS) generation and antioxidant defense mechanisms, plays a pivotal role in cancer treatment [31]. Modulating oxidative stress pathways holds promise as a therapeutic strategy, as it can sensitize cancer cells to chemotherapy and radiation therapy while mitigating damage to normal tissues [31]. 

**Figure 4:**
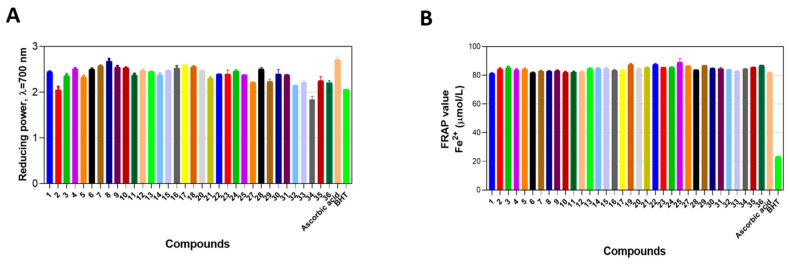
The (4-hydroxyphenyl)amino)propanoic acid derivatives shows promising activity. Panel (**A**) demonstrates the antioxidant screening data obtained by using employed ferric ion transformation assay, while panel (**B**) demonstrates the antioxidant activity of compounds **1**–**36** using ferric ion reduction power (FRAP) assay. The data are expressed as mean ± standard deviation of tree experimental replicas.

Therefore, after characterizing the in vitro cytotoxic properties of compounds **1**–**36**, we then aimed to characterize their antioxidant activity. To do so, we employed ferric ion transformation, ferric ion reduction assay, as well as 1,1-Diphenyl-2-picrylhydrazyl (DPPH) radical scavenging assays, where we systematically characterized the antioxidant properties of (4-hydroxyphenyl)amino)propanoic acid derivatives **1**–**36** (Figure 4A,B) [32]. 

The ferric ion (Fe^3+^) reducing antioxidant power of compounds **1**–**36** was evaluated and compared to ascorbic acid and the commercial antioxidant butylated hydroxytoluene (BHT). In this assay, ascorbic acid exhibited the highest antioxidant activity and was used as a control.

Among the tested compounds, compound **8** exhibited the most potent reducing capability, closely followed by compounds **17**, **7**, and **19**. Compounds **8** and **17**, both hydrazones, demonstrated superior antioxidant activity, suggesting that the presence of azomethine groups significantly enhances their reducing power. Similarly, compound **7**, another hydrazone, showed high activity, indicating that hydrazone groups are crucial for strong ferric ion reducing capabilities. Compound **19**, an oxadiazole, also displayed promising antioxidant activity, implying that its heterocyclic structure containing nitrogen and oxygen atoms contributes to its in vitro ferric ion reducing properties.

Subsequently, compounds **1**, **3**, **4**, **5**, **6**, **9**, **10**, **11**, **12**, **13**, **14**, **15**, **16**, **20**, **21**, **22**, **23**, **24**, **25**, **27**, **28**, **29**, **30**, **31**, **32**, **33**, **35**, and **36** exhibited varying degrees of reducing activity, dependent on their respective structural substituents. Many of these compounds are hydrazones, indicating a general trend of robust antioxidant activity within this chemical class, which is highly dependent on the presence of specific structural substituents (Figure 4A). Finally, similar results were observed when compounds **1**–**36** were analyzed using the FRAP assay. These compounds demonstrated significantly higher antioxidant activity in the FRAP assay compared to the BHT control (*p* < 0.05) (Figure 4B). 

After characterizing the antioxidant activity using iron-based assays, we further evaluated the antioxidant properties of compounds **1**–**36** using the DPPH radical scavenging assay.

**Figure 5:**
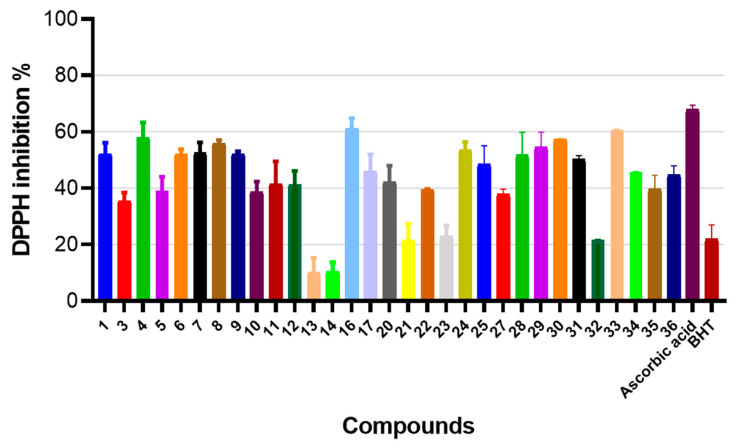
The (4-hydroxyphenyl)amino)propanoic acid derivatives **1**–**36** demonstrate strong antioxidant activity in DPPH radical scavenging assay. The data are expressed as mean ± standard deviation of three experimental replicas.

The results of this investigation revealed (Figure 5) that compounds bearing the 2,5-dimethyl-1*H*-pyrrol-1-yl (compound **16**, 61.2%), 3,4,5-trimethoxybenzylidene (compound **33**, 60.6%), dimethyl 3,3’-4-hydroxyphenyl (compound **4**, 57.9%), 4-chlorobenzylidene (compounds **30**, 57.4%, and **8**, 55.8%), 4-nitrobenzylidene (compounds **29**, 54.4%, and **7**, 52.5%), and *N*’-thiophen-3-ylmethylene (compound **24**, 53.6%) fragments possessed a high DPPH radical scavenging ability compared to the commercial antioxidant BHT (22.0%). Compounds **32** (21.6%), **14** (10.4%), and **13** (10.2%) demonstrated the lowest DPPH inhibition in comparison with BHT (22.0%).

## 3. Discussion

This study demonstrates that 3-((4-hydroxyphenyl)amino)propanoic acid derivatives could be further explored as novel scaffolds for developing compounds with anticancer and antioxidant properties. 

The 3-((4-hydroxyphenyl)amino)propanoic acid derivatives could be considered as promising pharmacophores in medicinal chemistry due to their favorable chemical properties, synthetic versatility, and the feasibility of incorporating various aromatic or heterocyclic substitutions [23]. These phenolic scaffolds contain a hydroxyl group at the para position on the phenyl ring, enhancing aqueous solubility and enabling robust hydrogen bonding interactions crucial for high-affinity target binding [33,34]. Additionally, the presence of the amino group adjacent to the propanoic acid moiety allows for the formation of amide bonds and incorporation of other bioactive modifications, facilitating diverse conjugation strategies [35,36,37]. The synthetic flexibility of 3-((4-hydroxyphenyl)amino)propanoic acid derivatives permits the introduction of various heterocyclic structures, such as imidazoles, pyridines, and triazoles, which can be tailored to optimize biological activity and specificity [23].

The 3-((4-hydroxyphenyl)amino)propanoic acid derivatives **1**–**36**, previously synthesized by our group, exhibit significant synthetic versatility, achievable through one- or two-step reactions utilizing commercially available building blocks [23]. This facile synthesis pathway underscores the practicality of these derivatives for large-scale production and further structural modifications. The resulting compounds have demonstrated favorable antimicrobial activity against multidrug-resistant bacterial and fungal pathogens [23]. This activity suggests that these derivatives can effectively target multiple cellular mechanisms in a structure-dependent manner. 

Various amino-acid-based derivatives have been previously investigated as novel therapeutic candidates targeting various diseases, including cancer [37,38]. Transformed cells have profound energetic requirements and thus an increased need for amino acids for the regulation of metabolic needs and protein production. Previous studies have shown that unnatural (xenogenic) amino acid derivatives possess strong anticancer activity via mitochondria-targeting pathways [38]. Further studies that focused on novel amino acid and dipeptide derivatives of neocryptolepine successfully demonstrated promising anticancer activity of synthesized compounds both in vitro and in vivo, demonstrating that the amino-acid-based approach could be further explored as a new chemotherapeutic strategy to target various neoplasms [39]. 

In our study, we successfully demonstrated that 3-((4-hydroxyphenyl)amino)propanoic acid derivatives, containing a phenolic group, exhibit structure-dependent anticancer activity against A549 non-small cell lung cancer cells. The most promising compound, **20**, containing a 2-furyl substituent, demonstrated selectivity towards cancerous cells using non-transformed Vero cell lines. Additionally, compound **20** reduced A549 cell migration, suggesting that 3-((4-hydroxyphenyl)amino)propanoic acid derivatives containing a 2-furyl substituent could be further explored for the generation of novel sub-libraries to enhance anticancer activity.

Oxidative stress, characterized by an imbalance between reactive oxygen species (ROS) generation and antioxidant defense mechanisms, plays a pivotal role in cancer treatment [40]. Modulating oxidative stress pathways holds promise as a therapeutic strategy, as it can sensitize cancer cells to chemotherapy and radiation therapy while mitigating damage to normal tissues [38,40]. The phenolic group within the 3-((4-hydroxyphenyl)amino)propanoic acid derivatives potentially confers significant antioxidative potential, which is important for modulating oxidative stress pathways in cancer treatment or synergizing cancer cells to chemotherapeutics [41,42]. The hydroxyl group in the phenolic structure can donate hydrogen atoms to neutralize ROS, thereby reducing oxidative damage to cellular components [43]. Additionally, the amino group adjacent to the phenolic ring enhances the electron-donating capacity, further stabilizing the phenoxyl radical formed after ROS scavenging [41,42]. These properties could make the 3-((4-hydroxyphenyl)amino)propanoic acid derivatives promising chemotherapeutic candidates with antioxidative activity, capable of protecting normal tissues while sensitizing cancer cells to chemotherapy and radiation therapy. In our work, we have demonstrated that 3-((4-hydroxyphenyl)amino)propanoic acid derivatives harbor antioxidant activity, suggesting that 3-((4-hydroxyphenyl)amino)propanoic acid could serve as a novel scaffold with both anticancer and antioxidant activities.

Collectively, this study shows that 3-((4-hydroxyphenyl)amino)propanoic acid could serve as a novel scaffold for the development of candidates with both anticancer and antioxidative activity. Compound **20**, containing a 2-furyl substituent, could serve as a starting point for hit-to-lead optimization.

## 4. Materials and Methods

### 4.1. Test Compounds and Screening Library Preparation

The synthesis and characterization of compounds **1**–**36** were previously described by Kavaliauskas et al. [23]. For this study, compounds (Table 1) obtained from the corresponding author were dissolved in hybridoma-grade dimethyl sulfoxide (Millipore, Sigma, Burlington, MA, USA) to prepare stock solutions at concentrations of 10–25 mg/mL. The dissolved compounds were then manually dispensed into deep 96-well plates, sealed, and stored at −80 °C until the day of the experiment. For the in vitro anticancer activity screening, the compounds were thawed at room temperature, protected from light, and the aliquots were diluted in complete cell culture media to achieve a final concentrations of 100 µM and used for the in vitro assays. 

### 4.2. Cell Lines and Culture Conditions

The A549 non-small cell human lung carcinoma cells (ATCC CCL-185) and Vero African green monkey kidney cells (ATCC CCL-81) were obtained from the American Type Culture Collection (Rockville, MD, USA). Cells were cultivated in Dulbecco’s Modified Eagle Medium/Nutrient Mixture F-12 (DMEM/F-12) (Gibco, Waltham, MA, USA), 10% fetal bovine serum (FBS) (Gibco, Waltham, MA, USA), 100 U/mL penicillin, and 100 μg/mL streptomycin (P/S) (Gibco, Waltham, MA, USA). Culturing conditions were maintained at 37 °C with a humidified atmosphere containing 5% CO_2_. The culture medium was refreshed every 2–3 days, and cells were passaged upon reaching 70–80% confluence.

### 4.3. Compound-Induced Cytotoxicity Evaluation Using MTT Assay

The viability of A549 cells following treatment with compounds or control agents was assessed using a commercial MTT (3-[4,5-dimethylthiazol-2-yl]-2,5-diphenyltetrazolium bromide) assay. Briefly, A549 or Vero cells were seeded in flat-bottomed 96-well microplates at a density of 1 × 10^4^ cells per well and incubated overnight to allow for attachment. The medium was then removed and compounds dissolved in cell culture medium were added to the wells. The cells were incubated for 24 hours at 37°C in a 5% CO_2_ atmosphere. Following incubation, 10 μL of Vybrant^®^ MTT Cell Proliferation Reagent (ThermoFisher Scientific, Waltham, MA, USA) was added to each well, and the cells were incubated for an additional 4 hours. After this period, the medium was removed, and the resulting formazan crystals were dissolved in 100 μL of DMSO. Absorbance was measured at 570 nm using a microplate reader (Multiscan, ThermoFisher Scientific, Waltham, MA, USA). The percentage of A549 or Vero cell viability was calculated using the following formula: ([*AE* − *AB*]/[*AC* − *AB*])×100%, where *AE*, *AC*, and *AB* represent the absorbance of the experimental samples, untreated control samples, and blank controls, respectively. All experiments were conducted in triplicate.

### 4.4. Cell Migration Assay

A549 cells were plated at a density of 2.5 × 10^5^ cells/well on the 24 well plates. After 48 h, when the cells had reached 100% confluence, wounds were made in the monolayer with non-barrier autoclaved 200 µL tips. The compounds were added to reach 100 µM concentration and were incubated for 24 h. After incubation, the media with compounds were removed; cells were washed twice with DPBS; a fresh, compound-free medium was added; and cells were incubated for an additional 24 h to facilitate wound closure. The percentage of wound healing was calculated by measuring the wound closure diameter by using Image J (Version 1.51 23 April 2018) [24] and normalizing the percentage of wound closure to untreated control. 

### 4.5. Determination of the Antioxidant Activity

#### 4.5.1. Ferric ion (Fe^3+^) Reducing Antioxidant Power Determination Assay 

Test compounds **1**–**36** at a concentration of 10 mM in 0.5 mL of DMSO were mixed with 1.25 mL of phosphate buffer (0.2 M, pH 6.6) and 1.25 mL of potassium ferricyanide [K_3_Fe(CN)_₆_] (1%) (Millipore, Sigma, Burlington, MA, USA). The mixture was incubated at 50 °C for 20 mins. After incubation, 1.25 mL aliquots of trichloroacetic acid (10%) were added and the mixture was centrifuged at 9000 rpm for 10 min. The supernatant (1.25 mL) was mixed with 1.25 mL of distilled water and 0.25 mL of FeCl_3_ (0.1%), and the absorbance was measured at 700 nm using a spectrophotometer (Shimadzu, Kyoto, Japan). Butylhydroxytoluene (BHT) and ascorbic acid were used as controls. All experiments were performed in triplicate. 

#### 4.5.2. Ferric Reducing Antioxidant Power Assay

The reducing properties of the compounds were investigated using the FRAP assay. Briefly, the FRAP reagent was prepared by combining 2.5 mL of 10 mM TPTZ (2,4,6-tripyridyl-*s*-triazine) solution in 40 mM HCl, 2.5 mL of 20 mM FeCl_3_, and 25 mL of acetate buffer (0.3 M, pH 3.6) (Millipore, Sigma, Burlington, MA, USA). Then, 100 µL of the compounds (10 mM) were mixed with 3 mL of the FRAP reagent. The absorbance of the reaction mixture was measured spectrophotometrically at 593 nm. 

#### 4.5.3. 1,1-Diphenyl-2-picrylhydrazyl (DPPH) Radical Scavenging Assay

The free-radical-scavenging activity of the novel compounds was measured using the DPPH assay. Briefly, a 10 mM solution of compounds **1**–**36** was prepared in DMSO. Subsequently, a 1 mM solution of DPPH in ethanol was prepared, and 1 mL of this solution was added to the solutions of the analyzed compounds. The mixture was vigorously stirred and allowed to stand at room temperature for 20 min. The absorbance of the reaction mixture was then measured at 517 nm using a UV-1280 spectrophotometer (Shimadzu, Kyoto, Japan). All experiments were performed in triplicate.

### 4.6. Statistical Analysis

The data are expressed as mean ± SD values from three separate experiments unless stated otherwise. The statistical significance was determined using a one-way ANOVA test. Data were considered significant when *p* < 0.05.

## 5. Conclusions

This study successfully demonstrated that 3-((4-hydroxyphenyl)amino)propanoic acid can serve as a novel scaffold for developing compounds with promising anticancer and antioxidant activities. This study provides an early preclinical foundation for the development of novel candidates for further preclinical evaluation using laboratory models. During this study, we identified the most promising derivative, 3,3′-((4-hydroxyphenyl)azanediyl)bis(*N*′-(furan-2-ylmethylene)propanehydrazide), compound **20**, which exhibits potent anticancer and antioxidant activities. This compound could be further explored for hit-to-lead optimization or serve as a starting point for the generation of compound-**20**-based sub-libraries. Further studies are needed to better understand the safety, tolerability, and in vivo properties of 3-((4-hydroxyphenyl)amino)propanoic acid derivatives, particularly compound **20**.

## Figures and Tables

**Table 1:** The representation of 3-((4-hydroxyphenyl)amino)propanoic acid derivatives used in this study.

No.	Compound	No.	Compound
**1**	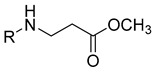	**19**	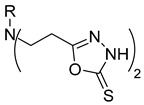
**2**	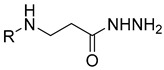	**20**	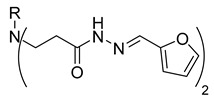
**3**	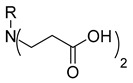	**21**	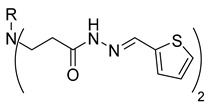
**4**	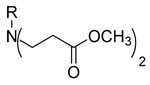	**22**	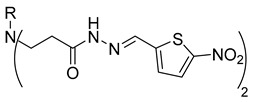
**5**	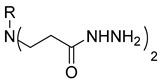	**23**	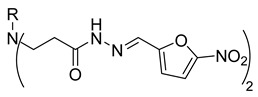
**6**	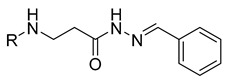	**24**	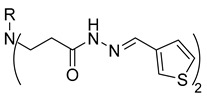
**7**	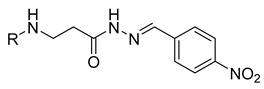	**25**	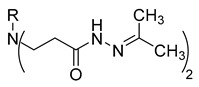
**8**	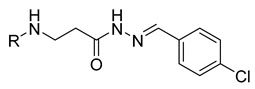	**26**	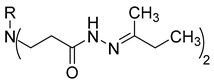
**9**	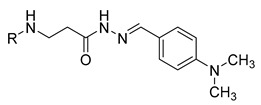	**27**	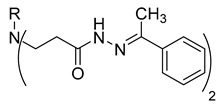
**10**	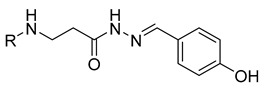	**28**	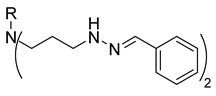
**11**	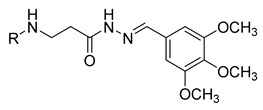	**29**	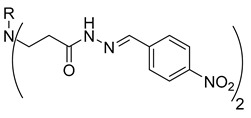
**12**	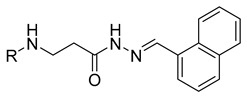	**30**	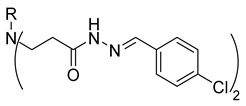
**13**	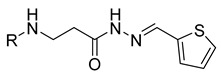	**31**	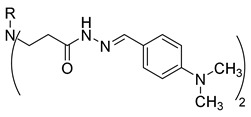
**14**	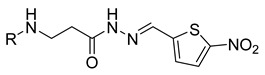	**32**	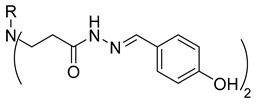
**15**	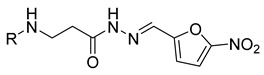	**33**	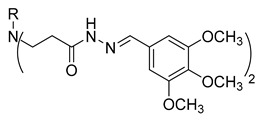
**16**	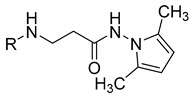	**34**	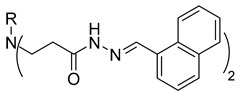
**17**	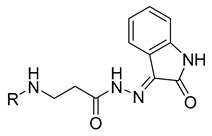	**35**	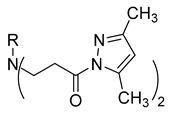
**18**	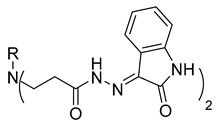	**36**	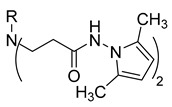
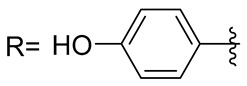			

## Data Availability

All data generated during this study are provided in the manuscript. Compounds are available by request to corresponding author.
